# Diagnostic Modalities Used in Diagnosing Gastroparesis: A Clinical Review

**DOI:** 10.7759/cureus.30540

**Published:** 2022-10-21

**Authors:** Haider Ghazanfar, Nishant Allena, Nismat Javed, Deny Ponnachan, Sanjana Narasimhadevara, Thrupthi Komadur, Ali Ghazanfar, Trishna Acherjee, Harish Patel

**Affiliations:** 1 Internal Medicine, BronxCare Health System, Bronx, USA; 2 Internal Medicine, SUNY Downstate, Brooklyn, USA; 3 Internal Medicine, Federal Medical and Dental College, Islamabad, PAK

**Keywords:** paracetamol absorption, gastroparesis in critical care, gastric emptying of radiopaque markers, 13 c breath test, electrogastrography, a gastrointestinal failure score, gastric emptying scintigraphy, wireless motility capsule, gastroparesis

## Abstract

Gastroparesis is associated with abnormal gastric motility characterized by delayed gastric emptying without any obvious mechanical gastric outlet obstruction or blockage. Gastroparesis is associated with significant morbidity and mortality. It is pertinent to make a timely diagnosis of gastroparesis so that prompt treatment can be initiated. The purpose of this clinical review article is to help the internist and the primary care providers to get a better idea of various diagnostic modalities used in diagnosing gastroparesis. We have also discussed the advantages and disadvantages of various diagnostic modalities based on the latest evidence.

## Introduction and background

Gastroparesis is associated with abnormal gastric motility characterized by delayed gastric emptying without any obvious mechanical gastric outlet obstruction or blockage. This results in the retention of gastric contents leading to several symptoms, commonly including nausea, vomiting, bloating, post-prandial fullness, and upper abdominal discomfort [1,2].

A systemic review of 13 studies showed that the prevalence of gastroparesis ranged from 13.8 to 267.7 per 100, 000 adults, and the incidence of gastroparesis was 1.9 - 6.3 per 100,000 person-years [3].

## Review

Pathophysiology and etiology of gastroparesis

The underlying pathophysiology of gastroparesis focuses on a dysfunctional nervous system in the gastric apparatus. As a result, many conditions can cause such dysfunction, including diabetes, medications (narcotics, antidepressants, dopamine agonists, octreotide, calcium channel blockers), post-viral syndromes (Rotavirus, Norwalk virus), neurologic diseases (Parkinson's disease, multiple sclerosis, autoimmune disorders, spinal cord injury), post-surgical conditions, infiltrative diseases and connective tissue diseases like amyloidosis and scleroderma [4, 5]. The most common cause by far is idiopathic. Contributing factors of gastroparesis include hyperglycemia as studies have shown that acute changes in glucose can accelerate or delay emptying [6]. Enteric nervous system abnormalities have also been implicated. Plourde et al. showed that decreased neuronal nitric oxide synthase in rats secondary to drugs or diseases affected gastric motility [7]. Interstitial cells of Cajal (ICC) play an important role in nitric oxide-dependent signal transduction in the gastrointestinal tract and the loss of these cells leads to the impaired peristaltic reflex of the intestine. Diabetes mellitus has a strong association with loss of ICC. Some chronic diseases can cause oxidative stress leading to a loss of anti-oxidant protection, such as heme oxygenase- 1 due to upregulation. A possible reversal of this loss can decrease the delay in gastric emptying as revealed in a study [8]. 

According to a study conducted from 1995 to 2004, gastroparesis was attributed to a 53% increased risk of diabetes-related hospitalizations [9]. This condition may also increase the risk of other diabetes-related complications. For example, patients with diabetes who experienced symptomatic gastroparesis were more likely to develop cardiovascular disease (19.2 vs. 6.4%, P <0.05), hypertension (63 vs. 43%, P = 0.005), and retinopathy (33 vs. 11.7%, P <0.001) [10]. Complications of diabetic gastroparesis include esophagitis, Mallory-Weiss tear, electrolyte disturbances, bezoar formation, and hyperglycemic emergencies [11]. Gastroparesis can also be attributed as a complication of several types of malignancies including gall bladder, gastric, esophageal, pancreatic, and lung cancers. This cause is relatively underdiagnosed and the pathogenesis is not fully understood. It could be attributed to malignant infiltration of the autonomic nervous system, and paraneoplastic dysmotility with autoantibody-mediated destruction of the enteric nervous system. Sachdeva S, Ghosal et al have demonstrated the increased incidence of gastroparesis in patients with cholangiocarcinoma [12]. 

Parkinson's disease is also an underrecognized cause of gastroparesis. It is believed that the accumulation of α-synuclein deposits could lead to damage in the enteric neuronal network leading to an impairment in gastric motility [13].

It is essential to obtain a good history, both clinical and medication history to accurately diagnose and if possible determine the underlying cause of gastroparesis. Some physical exam findings suggestive of gastroparesis include abdominal distension, epigastric tenderness, succussion splash, halitosis, and orthostatic and postprandial hypotension [11]. Although laboratory tests might aid in the diagnosis, imaging studies are of immense importance in this regard.

**Figure 1:**
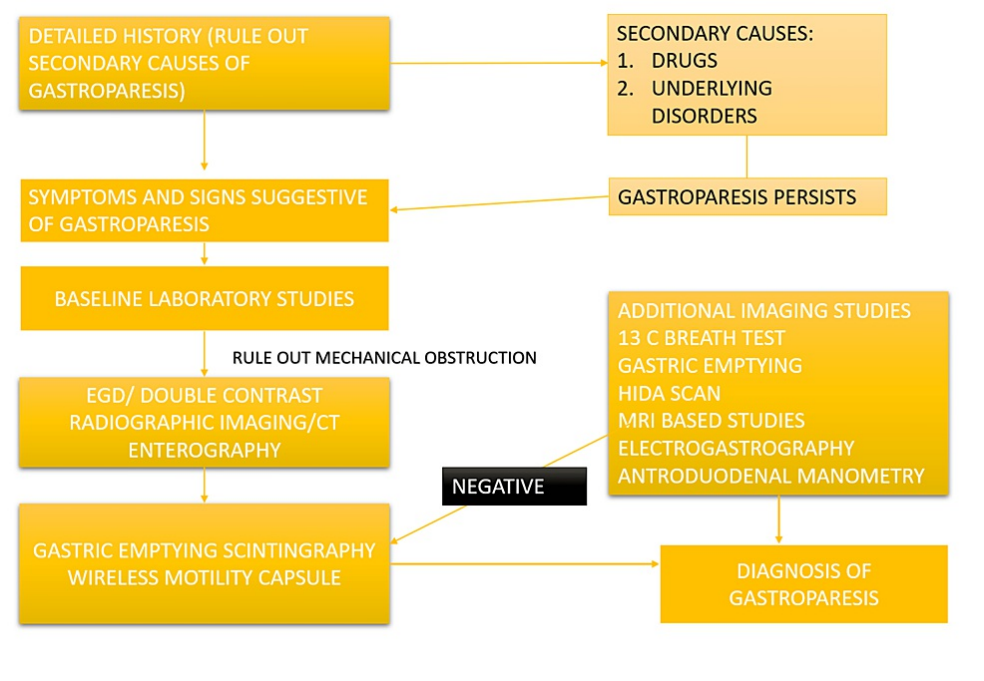
Clinical Flow Chart for Diagnosing Gastroparesis. Image Credits: Haider Ghazanfar, Nishant Allena, Nismat Javed

A flow chart for the assessment of gastroparesis has been presented in Figure 1.

Ruling out mechanical obstruction

The initial step in imaging is to rule out mechanical obstruction prior to obtaining studies. The mechanical obstruction can be diagnosed by esophagogastroduodenoscopy. Some patients with retained food on endoscopy might have normal scintigraphic emptying, suggesting relatively preserved postprandial antral motility to triturate and empty a digestible meal (during scintigraphy) but abnormal inter-digestive antral motility that impairs emptying from the stomach between meals of particles larger than 2 mm in size [14]. Other studies include double-contrast upper gastrointestinal radiography [15]. It can help demonstrate the presence of a hiatal hernia or any obstruction of the small intestine. It can aid in, but not make a definitive diagnosis of gastroparesis. The suggestive radiological features include gastric dilatation, retained gastric content, delayed gastric emptying of barium, and decreased or absent peristalsis [16]. Lastly, computed tomography enterography combines oral and IV contrast ("dye") with high-resolution x-ray imaging. magnetic resonance enterography uses an oral contrast dye to highlight the small intestine [17]. These are additional tests that help rule out any mass, ulcer, or stricture present in the gastrointestinal tract. 

Once mechanical obstructions have been ruled out, the next step to obtaining a definitive diagnosis of gastroparesis is to assess gastric motility. 

Gastric emptying scintigraphy

Gastric scintigraphy is a test of choice in diagnosing gastroparesis. By measuring the amount of radioactivity in the stomach at various time points, gastric scintigraphy is more sensitive to solid food emptying compared to liquids since liquid emptying remains normal despite advanced disease [11,18]. Prokinetic medications that delay gastric emptying must be held for at least 48 hours before the test [19]. Tight glycemic control should be achieved to minimize interference with gastric emptying. Current guidelines recommend low-fat egg white meal, labeled with 0.5 mCi tech 99 m sulfur colloid radioisotope within 10 mins and imaging immediately followed by imaging at one, two, and four hours [20]. Ideally, around 40-90% of food remains at one hour, 30-60% at the end of two hours, and 0- 10% at four hours. Gastric emptying is considered delayed if there is greater than 60% retention at 2h or 10% retention at 4h. In a study conducted in Malaysia focusing on 13 patients, one patient showed rapid gastric emptying, three patients demonstrated delayed emptying in the early phase with normal gastric retention at 4h, and six patients reported delayed gastric emptying at 4h of the study [21]. Of the six patients, four patients had mildly delayed gastric emptying (11% to 20% retention). The majority of the patients with nausea and vomiting (75%) had an abnormal scan [21]. In another study exploring gastric per-oral endoscopic myotomy as a potential therapy, gastric emptying scintigraphy was used to assess the retention index or propagation ratios of proximal and distal areas of gastroparesis very accurately [22].

Wireless motility capsule 

The wireless motility capsule (WMC) is used to monitor the transit of food from the stomach to the small bowel by detecting changes in pH from an acidic to an alkaline environment [23, 24]. As with gastric scintigraphy, medications altering gastric motility and that surpass gastric acid are to be stopped 48 hours and 72 hours prior to the procedure respectively. The patient consumes a nutrient meal along with the WMC capsule with pH data interpreted on the receiver worn by the patient. Gastric emptying is defined by the time from ingestion of the capsule to change in pH by 4 units ideally within five hours. Delayed emptying is considered when the change in pH happens > 6hrs. WMC had a sensitivity of 59 to 86 % and a specificity of 64 to 81% for gastric paresis [25]. In a study of 107 patients with gastroparesis of which 75 were diabetic, a wireless motility capsule was used to determine associations with symptomatology. A negative significant correlation was found between colonic motility index (rs = −0.34, p = 0.012) and symptomatology score. Negative correlations between the mean pressure of the colon (rs = −0.37, p = 0.007) and upper abdominal pain were also present [26]. In another study conducted in 2018 on 23 gastroparetics, wireless motility capsules revealed that the motility parameters fail to significantly increase in the small bowel suggesting neuropathic changes outside the stomach [27].

13 C breath test

13 C breath test also known as gastric emptying breath test uses stable isotope 13 C labeled substrates. 13C octanoic acid or 13C spirulina platensis is the most used substrate. It is also noninvasive and unlike scintigraphy does not use radiation exposure. The meal tagged with 13 C substrate passes through the stomach, into the duodenum where it is absorbed, metabolized in the liver, and exhaled by the lungs where it is measured [28]. The rate of gastric emptying of the 13 C substrate is measured by the breath excretion of 13CO2 by spectrometry. 13 C breathe test serves as an indirect way of measuring gastric emptying, dependent on normal bowel, liver, and lung function [29]. 13 C breathe test had about 80% specificity and sensitivity of 89%. 

The sensitivity and specificity of all the above-mentioned tests have been mentioned in Table 1.

**Table 1:** The sensitivity and specificity of diagnostic tests Reference: [23] [24] [28] [30] [31]

	Specificity (%)	Sensitivity (%)
Wireless motility capsule	59-86	64-81 (28)
13 C breath test	80	89 (29)
Gastric Scintigraphy @ 2H	20	100 (2)
Gastric Scintigraphy @ 4H	70	100 (2)
Gastric Emptying of radiopaque markers	97%	34%

Gastric emptying of radiopaque markers

Gastric emptying of radiopaque markers is a simple widely available inexpensive study that measures gastric emptying using fluoroscopy to assess the clearance of multiple small indigestible solid particles. A study done on 115 patients concluded that the sensitivity and specificity of this test were 34% and 97% respectively [30]. Compared to other diagnostic modalities it has low diagnostic reliability. Patients with high clinical suspicion of gastroparesis and a negative gastric emptying of radiopaque markers should undergo scintigraphy. Radiopaque markers were used in a study based on 45 patients with diabetes mellitus. It was affordable and available as compared to emptying studies [31].

Magnetic resonance imaging (MRI) based studies

MRI-based imaging is another promising way of diagnosing gastroparesis. Hayakawa et al. conducted a study on transplant patients that revealed an association between reduced velocity and prolonged gastric emptying. Gastric content volume ratios on MRI showed significant moderate positive correlations with gastric emptying parameters. There were no significant correlations between peristaltic wave frequency and gastric emptying parameters [32]. In another study by Ajaj et al, patients with gastroparesis showed a lower motility index compared with the reference volunteer group while the mean motility index of the patient group with pylorospasm was more than three times higher than that of the reference value of the volunteer group. However, the gastric motility index in the patient group with gastroparesis increased, and in the group, with functional pylorospasm/peptic pyloric stenosis, it decreased significantly after therapy [33].

Hepatobiliary iminodiacetic acid (HIDA)

In a specific subset of patients with Roux-en-Y gastric bypass, the caveat is to adopt imaging techniques that evaluate the bypassed stomach. One of such proposed techniques includes the HIDA scan as discussed in a case report by Tarakji et al. Hepatobiliary scintigraphy is a noninvasive dynamic study that can evaluate both functional gastroparesis and anatomic obstruction and leak pathology of the biliopancreatic limb after Roux-en-Y gastric bypass (RYGBP) [34].

Electrogastrography 

Electrogastrography (EGG) is a non-invasive technique that records gastric myoelectrical activity. It is suspected that 50 to 75% of patients with gastroparesis have at least one abnormality in the EGG [35]. A reduced percentage of normal slow waves decreased postprandial EGG dominant power and excessive gastric dysrhythmia are the common abnormalities seen in EGG in patients with gastroparesis [35]. Currently, it is only used on patients enrolled in clinical trials and further studies are needed to establish its clinical application. In a study conducted in 1993 about the efficacy of cisapride, EGG was used. Twelve of 14 patients had abnormal baseline electrogastrograms that improved after six months of cisapride, in both idiopathic and diabetic etiologies [36].

The advantage and disadvantage of each of the above modality have been mentioned in Table 2.

**Table 2:** The advantages and disadvantages of diagnostic tests for gastroparesis Reference: [32], [33], [34]

MODALITY	ADVANTAGES	DISADVANTAGES
Gastric Emptying Scintigraphy	More sensitive to solid food emptying Real-time imaging with a specific number of hours	Require a fasting period, cannot be used in emergencies Optimal control of blood glucose levels in diabetic patients is needed. Anaphylaxis to an ingested meals is Contraindicated in cases of dysphagia, any oropharyngeal or esophageal stricture, or functional abnormality.
Wireless Motility Capsule	Accurate at evaluating gastric and transit pH in critically-ill patients Non-invasive	Medications altering gastric acidity will tamper results Contraindicated in cases of dysphagia, any oropharyngeal or esophageal stricture, or functional abnormality.
13 C Breath Test	Non-invasive and does not require radioactive materials	High chances of false positives and false-negatives
Gastric Emptying of Radiopaque Markers	Inexpensive and widely available Can be used for screening and exclusion	Low diagnostic reliability and requires supplementation with other methods
Magnetic Resonance Imaging based studies	Non-invasive	Has been studied in specific subgroups
Hepatobiliary iminodiacetic acid	Non-invasive	Has been studied in a specific subgroup
Electrogastrography	Non-invasive	Requires further investigation to determine use in clinical setups

Antroduodenal manometry

Antroduodenal Manometry is an invasive technique that records stomach and duodenal motility. It has aided in making a diagnosis with 8-15% of patients [37] with symptoms of unexplained nausea, vomiting, and abdominal pain. It is particularly useful in diagnosing suspected small intestinal pseudo-obstruction. It is carried out with the help of either a solid-state catheter or a water-based catheter and more recently high resolution manometry has been shown to give details on small bowel & pyloric pressure at various stages of contraction [38].

Diagnosing gastroparesis in a critical care setting

Gastroparesis is an important factor that influences morbidity and mortality in critically ill patients. Sixty percent of critically ill patients have experienced some form of GI motility. It can lead to complications like malnutrition, gastroesophageal reflux, and SIBO. A gastrointestinal failure (GIF) score, which is a combination of intra-abdominal pressure and feeding intolerance was shown to serve as an independent risk factor for estimating mortality in the ICU in a study conducted by Reintam et al [39]. 

Gastric emptying scintigraphy is a time-consuming test and is not widely used in practice for diagnosing gastroparesis in critically ill patients [40]. Paracetamol absorption is another technique that can be used for gastric emptying assessment [41]. Currently, there is very limited data on it and additional research is needed for further standardization. 

## Conclusions

A delay in the diagnosis of gastroparesis is associated with significant morbidity and mortality. Primary care providers and internists should be aware of various diagnostic modalities that can be used for diagnosing gastroparesis. Each diagnostic modalities have its own advantage and disadvantage. Physicians should use a patient-centered approach in choosing a diagnostic modality for the diagnosis of gastroparesis.

## References

[1] (2022). Gastroparesis. https://gi.org/topics/gastroparesis/.

[2] Camilleri M, Parkman HP, Shafi MA, Abell TL, Gerson L (2013). Clinical guideline: management of gastroparesis. Am J Gastroenterol.

[3] Dilmaghani S, Zheng T, Camilleri M (2022). Epidemiology and healthcare utilization in patients with gastroparesis: a systematic review. Clin Gastroenterol Hepatol.

[4] Moshiree B, Potter M, Talley NJ (2019). Epidemiology and pathophysiology of gastroparesis. Gastrointest Endosc Clin N Am.

[5] Ye Y, Yin Y, Huh SY, Almansa C, Bennett D, Camilleri M (2022). Epidemiology, etiology, and treatment of gastroparesis: real-world evidence from a large US National claims database. Gastroenterology.

[6] Schvarcz E, Palmér M, Aman J, Lindkvist B, Beckman KW (1993). Hypoglycaemia increases the gastric emptying rate in patients with type 1 diabetes mellitus. Diabet Med.

[7] Plourde V, Quintero E, Suto G, Coimbra C, Taché Y (1994). Delayed gastric emptying induced by inhibitors of nitric oxide synthase in rats. Eur J Pharmacol.

[8] Choi KM, Kashyap PC, Dutta N (2010). CD206-positive M2 macrophages that express heme oxygenase-1 protect against diabetic gastroparesis in mice. Gastroenterology.

[9] Wang YR, Fisher RS, Parkman HP (2008). Gastroparesis-related hospitalizations in the United States: trends, characteristics, and outcomes, 1995-2004. Am J Gastroenterol.

[10] Hyett B, Martinez FJ, Gill BM, Mehra S, Lembo A, Kelly CP, Leffler DA (2009). Delayed radionucleotide gastric emptying studies predict morbidity in diabetics with symptoms of gastroparesis. Gastroenterology.

[11] Waseem S, Moshiree B, Draganov PV (2009). Gastroparesis: current diagnostic challenges and management considerations. World J Gastroenterol.

[12] Sachdeva S, Ghoshal U C, Saraswat VA, Das K, Misra A (2006). Gastroduodenal dysmotility in patients with gallbladder carcinoma: frequency of occurrence and clinical importance. Natl Med J India.

[13] Soliman H, Coffin B, Gourcerol G (2021). Gastroparesis in parkinson disease: pathophysiology, and clinical management. Brain Sci.

[14] Camilleri M, Malagelada JR (1984). Abnormal intestinal motility in diabetics with the gastroparesis syndrome. Eur J Clin Invest.

[15] Freeny PC (1979). Double-contrast gastrography of the fundus and cardia: normal landmarks and their pathologic changes. AJR Am J Roentgenol.

[16] Szarka LA, Camilleri M (2019). Evaluation of patients with suspected gastroparesis. Gastrointest Endosc Clin N Am.

[17] Parkman HP, Hasler WL, Fisher RS (2004). American Gastroenterological Association technical review on the diagnosis and treatment of gastroparesis. Gastroenterology.

[18] Camilleri M, Zinsmeister AR, Greydanus MP, Brown ML, Proano M (1991). Towards a less costly but accurate test of gastric emptying and small bowel transit. Dig Dis Sci.

[19] Farrell MB (2019). Gastric emptying scintigraphy. J Nucl Med Technol.

[20] Abell TL, Camilleri M, Donohoe K (2008). Consensus recommendations for gastric emptying scintigraphy: a joint report of the American Neurogastroenterology and Motility Society and the Society of Nuclear Medicine. Am J Gastroenterol.

[21] Rohani MF, Zanial AZ, Nagaratnam P, Gew LT, Mutalib NA, Hassan SZ (2021). Radionuclide gastric emptying scintigraphy in patients with suspected gastroparesis in Hospital Kuala Lumpur: A preliminary experience. Med J Malaysia.

[22] Spandorfer R, Zhu Y, Abdelfatah MM (2020). Proximal and distal gastric retention patterns in gastroparesis and the impact of gastric per-oral endoscopic myotomy: a retrospective analysis using gastric emptying scintigraphy. J Nucl Med Technol.

[23] Camilleri M, Bharucha AE, di Lorenzo C, Hasler WL, Prather CM, Rao SS, Wald A (2008). American Neurogastroenterology and Motility Society consensus statement on intraluminal measurement of gastrointestinal and colonic motility in clinical practice. Neurogastroenterol Motil.

[24] Szarka LA, Camilleri M (2010). Stomach dysfunction in diabetes mellitus: emerging technology and pharmacology. J Diabetes Sci Technol.

[25] Zheng T, Camilleri M (2021). Management of gastroparesis. Gastroenterol Hepatol (N Y).

[26] Bekkelund M, Sangnes DA, Søfteland E (2021). Gastroparesis symptoms associated with intestinal hypomotility: an explorative study using wireless motility capsule. Clin Exp Gastroenterol.

[27] Surjanhata B, Brun R, Wilding G, Semler J, Kuo B (2018). Small bowel fed response as measured by wireless motility capsule: comparative analysis in healthy, gastroparetic, and constipated subjects. Neurogastroenterol Motil.

[28] Ghoos YF, Maes BD, Geypens BJ (1993). Measurement of gastric emptying rate of solids by means of a carbon-labeled octanoic acid breath test. Gastroenterology.

[29] van de Casteele M, Luypaerts A, Geypens B, Fevery J, Ghoos Y, Nevens F (2003). Oxidative breakdown of octanoic acid is maintained in patients with cirrhosis despite advanced disease. Neurogastroenterol Motil.

[30] Olausson EA, Brock C, Drewes AM (2013). Measurement of gastric emptying by radiopaque markers in patients with diabetes: correlation with scintigraphy and upper gastrointestinal symptoms. Neurogastroenterol Motil.

[31] Sangnes DA, Søfteland E, Teigland T, Dimcevski G (2019). Comparing radiopaque markers and 13C-labelled breath test in diabetic gastroparesis diagnostics. Clin Exp Gastroenterol.

[32] Hayakawa N, Nakamoto Y, Chen-Yoshikawa TF (2017). Gastric motility and emptying assessment by magnetic resonance imaging after lung transplantation: correlation with gastric emptying scintigraphy. Abdom Radiol (NY).

[33] Ajaj W, Goehde SC, Papanikolaou N, Holtmann G, Ruehm SG, Debatin JF, Lauenstein TC (2004). Real time high resolution magnetic resonance imaging for the assessment of gastric motility disorders. Gut.

[34] Tarakji AM, Morales F, Rovito P (2007). Hepatobiliary scintigraphy as a diagnostic modality for gastroparesis of the bypassed stomach after gastric bypass for morbid obesity. Obes Surg.

[35] Yin J, Chen JD (2013). Electrogastrography: methodology, validation and applications. J Neurogastroenterol Motil.

[36] Al Kafee A, Cilacı T, Kayar Y, Akan A (2022). Electrogastrography in patients with functional dyspepsia, joint hypermobility, and diabetic gastroparesis. Turk J Gastroenterol.

[37] Verhagen MA, Samsom M, Jebbink RJ, Smout AJ (1999). Clinical relevance of antroduodenal manometry. Eur J Gastroenterol Hepatol.

[38] Desipio J, Friedenberg FK, Korimilli A, Richter JE, Parkman HP, Fisher RS (2007). High-resolution solid-state manometry of the antropyloroduodenal region. Neurogastroenterol Motil.

[39] Reintam A, Parm P, Kitus R, Starkopf J, Kern H (2008). Gastrointestinal failure score in critically ill patients: a prospective observational study. Crit Care.

[40] Stojek M, Jasiński T (2021). Gastroparesis in the intensive care unit. Anaesthesiol Intensive Ther.

[41] Willems M, Quartero AO, Numans ME (2001). How useful is paracetamol absorption as a marker of gastric emptying? A systematic literature study. Dig Dis Sci.

